# Incidence and risk factors for chronic kidney disease in patients with congenital heart disease

**DOI:** 10.1007/s00467-021-05129-1

**Published:** 2021-05-25

**Authors:** Nai-Wen Fang, Yu-Chieh Chen, Shih-Hsiang Ou, Chun-Hao Yin, Jin-Shuen Chen, Yee-Hsuan Chiou

**Affiliations:** 1 Division of Pediatric Nephrology, Department of Pediatrics, Kaohsiung Veterans General Hospital, No 386, Dazhong 1st Rd, Zuoying Dist, Kaohsiung City, 813 Taiwan; 2 Division of Nephrology, Department of Internal Medicine, Kaohsiung Veterans General Hospital, Kaohsiung, Taiwan; 3 Department of Medical Education and Research, Kaohsiung Veterans General Hospital, Kaohsiung, Taiwan; 4 Department of Internal Medicine, Kaohsiung Veterans General Hospital, Kaohsiung, Taiwan; 5 School of Medicine, National Defense Medical Center, Taipei, Taiwan

**Keywords:** Children, Risk factors, Congenital heart disease, Chronic kidney disease

## Abstract

**Backgrounds:**

Chronic kidney disease (CKD) is underdiagnosed in children with congenital heart disease (CHD). Our aim was to study the incidence of CKD in CHD children and identify risk factors for CKD.

**Methods:**

CHD patients were enrolled from the Kaohsiung Veterans General Hospital database between 2010 and 2019. Patient age at enrollment was age at first visit to the hospital. The end of follow-up was marked by the last measurement of serum creatinine, urine protein-to-creatinine ratio (UPCR), or urine microalbumin-to-creatinine ratio (UACR) after enrollment, and only patients who underwent the aforementioned tests in 2 different years were included. Patients with an estimated glomerular filtration rate (eGFR) < 90 mL/min/1.73m^2^ were diagnosed as having CKD and were further classified into clinically recognized CKD (CR-CKD, defined as eGFR <60 mL/min/1.73m^2^, UPCR >0.5, or UACR >30 mg/g) and non-clinically recognized CKD (NCR-CKD). Their demographic data, CHD category, heart surgery types, medications, and contrast-related examinations during follow-up were collected.

**Results:**

The study included 359 CHD patients, of whom 167 (46.5%) developed CKD (18 patients with CR-CKD and 341 with NCR-CKD). Patients with CR-CKD were significantly older at enrollment than patients with NCR-CKD. Corrective heart surgery may be a protective factor for CKD. Furthermore, cyanotic heart disease, two or more image-related contrast exposures, and diuretic use may be associated with CKD.

**Conclusion:**

CHD patients have a high incidence of CKD. The early detection of CKD and prompt corrective heart surgery for CHD may be beneficial for kidney function.

**Graphical abstract:**

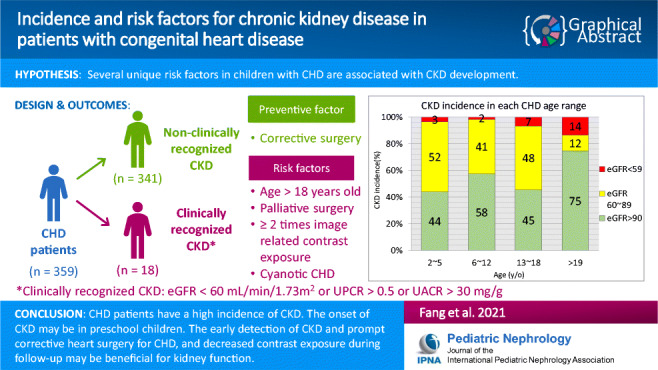

## Introduction

Congenital heart disease (CHD) is the most common birth defect in children. The survival rate of patients with CHD has improved with advances in surgical and medical care in the last few decades [1], and because more patients with CHD survive childhood into adulthood, many experience multiple noncardiac complications, with kidney dysfunction being the most prevalent. Nearly 50% of adults with CHD have an abnormal glomerular filtration rate [2], and 17% have significant albuminuria [3]. Both kidney dysfunction and albuminuria are closely linked to increased mortality [2, 3]. Numerous factors are related to the development of chronic kidney disease (CKD); some are nonmodifiable, such as ischemic insult caused by cardiopulmonary bypass during major surgery, hyperviscosity caused by uncorrected cyanotic heart disease, subsequent glomerular and tubular injuries, and various congenital anomalies of the kidney and urinary tract (CAKUT) related to syndromic CHD. Some modifiable risk factors for preventing further kidney injury include the use of contrast agents in cardiac imaging or cardiac catheterization and medications for controlling heart failure, such as angiotensin-converting enzyme inhibitors (ACEIs), angiotensin receptor blockers (ARBs), and diuretics. The aforementioned CKD risk factors may affect patients with CHD from childhood; however, early-stage CKD is insidious and often underdiagnosed in children. Early recognition and prompt treatment of CKD while monitoring patients with CHD may improve kidney outcomes. In the present study, we assessed the incidence of CKD in patients with CHD and identified modifiable risk factors for kidney dysfunction.

## Methods

### Study design: enrollment of patients with CHD, inclusion and exclusion criteria

Extracted from the Kaohsiung Veterans General Hospital (KSVGH) database, the data of 2172 patients with CHD were included in this study. CHD was defined using the *International Classification of Diseases, Ninth Revision* (ICD-9) codes 745, 746, and 747.0–747.4 or ICD-10 codes Q20–Q26. The KSVGH database contains the data of patients who visited KSVGH between 2010 and 2019, in which their primary diagnosis (both ICD-9 and ICD-10 codes), sex, height, weight, laboratory measurements, clinical interventions, and prescribed medications were recorded.

A patient’s age at enrollment was defined as the age when the patient first visited KSVGH between 2010 and 2019, and a patient’s end of follow-up was defined as the last test for their serum creatinine, urine protein-to-creatinine ratio (UPCR), or urine albumin-to-creatinine ratio (UACR). Patients were enrolled if they underwent serum creatinine, UPCR, or UACR testing in 2 different years during the study period, during which follow-up visits of the patients were recorded. According to the aforementioned criteria, 1352 patients were excluded. At enrollment, the exclusion criteria were as follows:

1. Patients with patent ductus arteriosus (PDA) and patent foramen ovale that closed spontaneously before they were 6 months old were excluded because these manifestations were more a physiological than a pathological process.
2. Patients with preexisting kidney disease, multiple anomalies, or confounding factors affecting kidney function calculation (such as short stature, failure to thrive, or prematurity) were excluded.
3. Patients younger than 2 years at the end of follow-up were excluded.

**Fig. 1:**
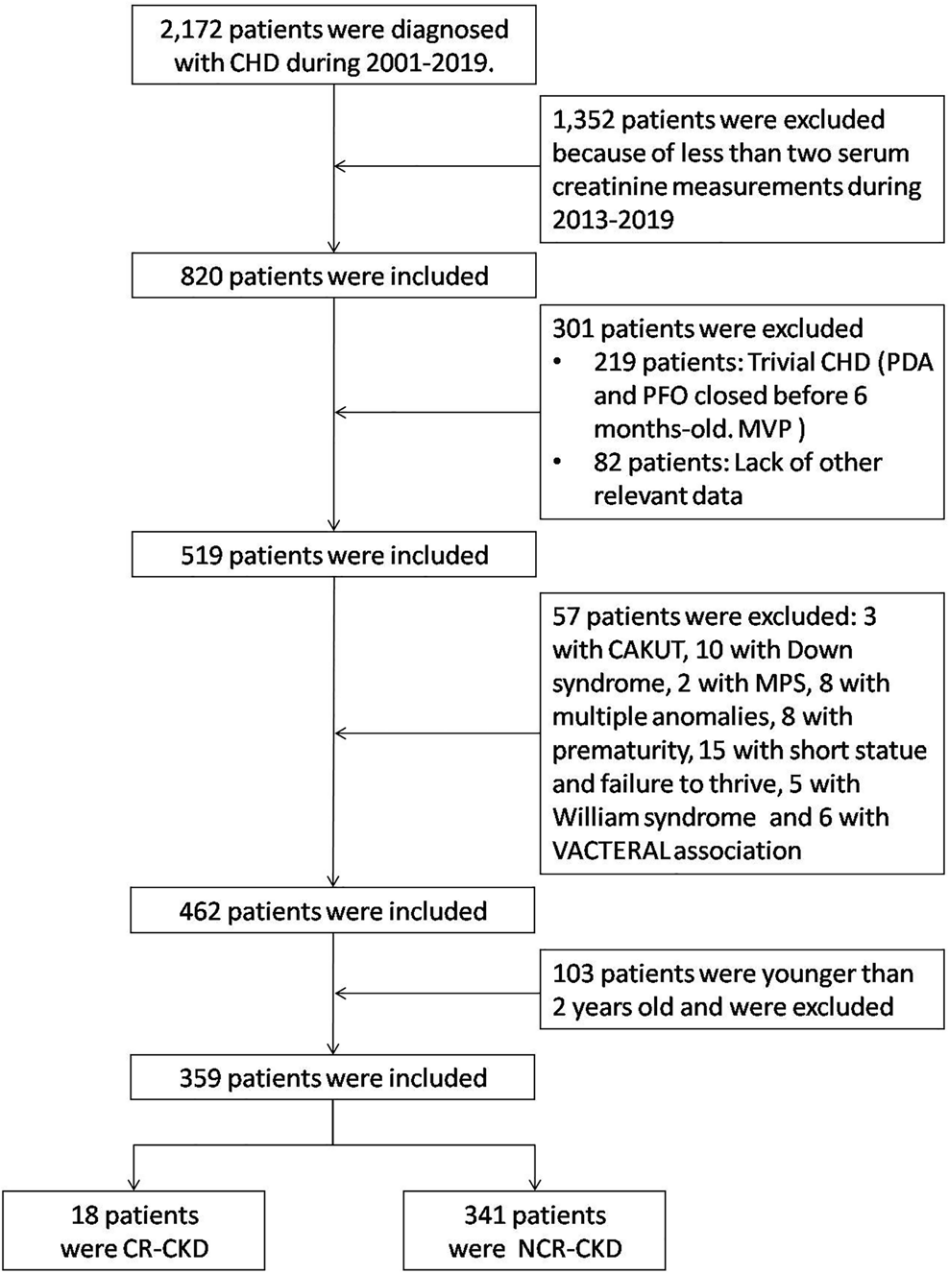
Study flow diagram. The evaluation comprised 359 patients with CHD. CAKUT congenital anomalies of the kidney and urinary tract, CHD congenital heart disease, CKD chronic kidney disease, MPS mucopolysaccharidosis, MVP mitral valve prolapse, PDA patent ductus arteriosus, PFO patent foramen ovale

In total, 359 patients were eligible for analysis (Fig. 1).

### Study protocol and definitions

Retrospectively, we recorded the incidence of CKD in patients with CHD, and classified patients into clinically recognized CKD (CR-CKD) and non-clinically recognized CKD (NCR-CKD). Variables upon enrollment and during the follow-up period in these two groups were compared. The incidence of patients with CKD was defined as patients who did not receive a diagnosis of CKD prior to enrollment and subsequently developed CKD during follow-up between 2010 and 2019. Patient age at CKD development was recorded and defined as the CKD onset age. A patient was diagnosed as having CKD when their estimated glomerular filtration rate (eGFR) was <90 mL/min/1.73 m^2^ in two consecutive tests. CR-CKD was defined if a patient fulfilled any of the following criteria: eGFR of <60 mL/min/1.73 m^2^, UPCR of >0.5, or UACR >30 mg/g [4]. eGFR was calculated using the modified Schwartz formula (eGFR (mL/min/1.73 m^2^) = (0.413 × height in cm)/serum creatinine in mg/dL) for patients aged 2 to 18 years, and the Modification of Diet in Renal Disease formula (eGFR (mL/min/1.73 m^2^) = 175 × (serum creatinine)^−1.154^ × (age)^−0.203^ × (0.742 if female) × (1.212 if African American) was used for patients older than 19 years. By the end of follow-up, 18 patients were classified as having CR-CKD and 341 patients were classified as having NCR-CKD.

Patients with underlying CHD were recorded upon enrollment, and if multiple CHD anomalies were noted in a patient, the most relevant CHD diagnosis for pathological cardiovascular circulation was recorded as indexed CHD. Patients with CHD were further classified as having cyanotic or acyanotic, PDA-dependent or independent, and simple or complex [5] CHD and, if they had been untreated, they were classified as developing right-sided or left-sided heart failure. Patients’ heart surgery history was reviewed at enrollment; patients were classified as having no need for surgery if their CHD was spontaneously cured or if intervention occurred without a cardiopulmonary bypass, such as through cardiac catheterization. For patients who received open-heart surgery, the surgery was classified as corrective if a complete correction of CHD occurred. However, if patients were under a staged approach to final correction or were precluded from complete correction by anatomic or patient factors, the surgery was classified as palliative. The Risk Adjustment for Congenital Heart Surgery (RACHS-1) score [6] for each surgery was recorded for comparison with the complexity and baseline operative risks.

#### Variables analyzed during study cohort

Patient age, sex, and Charlson comorbidity index score (CCIS) [7] were collected at enrollment. During follow-up, the medications (ACEIs, ARBs, beta-blockers, diuretics, digoxin, and anticoagulants) prescribed were recorded. The frequency of contrast-related imaging studies (computed tomography (CT) and magnetic resonance imaging (MRI)) and cardiac catheterizations in patients were noted.

#### Statistical analysis

All statistical analyses were performed using SPSS for Windows version 20 (SPSS Inc, Chicago, IL, USA). Comparisons among dichotomous variables were calculated using a Chi-square test or Fisher’s exact test. Continuous variables were expressed as mean ± standard deviation and compared using the one-way analysis of variance. Univariate logistic regression was then performed, and variables with a *P* value <0.1 were subject to multivariate logistic analysis. A backward stepwise selection process was applied to assess the influence of various factors for CKD development. The results of the comparison were expressed as *P* values. Statistical significance was set to *P* < 0.05.

## Results

### Overall characteristics of patients with CHD

Among the 359 patients with CHD, the most prevalent CHD was a ventricular septal defect (VSD; 20%), followed by tetralogy of Fallot (TOF; 14.7%), atrial septal defect (ASD; 11.1%), transposition of the great arteries (TGA; 7.2%), and pulmonary stenosis (PS; 5.6%). The mean age at enrollment was 6.6 years, and 195 (54%) of the enrollees were male. CCISs of zero were registered for 314 (86%) patients. Within the categories of CHD, 138 (38%) patients had cyanotic heart disease and 154 (43%) had severe CHD. In both categories, TOF, TGA, and pulmonary atresia were the most common CHDs. A total of 133 patients experienced PDA-dependent CHD, and 150 (42%) had CHD that would result in left-sided heart failure if left untreated. Overall, 149 (42%) patients with CHD did not require open-heart surgery; VSD, ASD, and PDA were the most prevalent CHDs among these patients. Of the remaining 210 (58%) patients with CHD, 156 (43%) received corrective heart surgery and 54 (15%) received palliative heart surgery. VSD repair was the most common corrective heart surgery, and a Blalock–Taussig shunt or the Fontan procedure for TOF were the most common palliative heart surgeries. The RACHS-1 score category for 210 surgeries were 11(5.2%), 126 (60%), 43 (20.4%), 29 (13.8%), and 1 (0.4%) surgery for categories 1, 2, 3, 4, and 6, respectively.

### CKD incidence and onset age

**Fig. 2:**
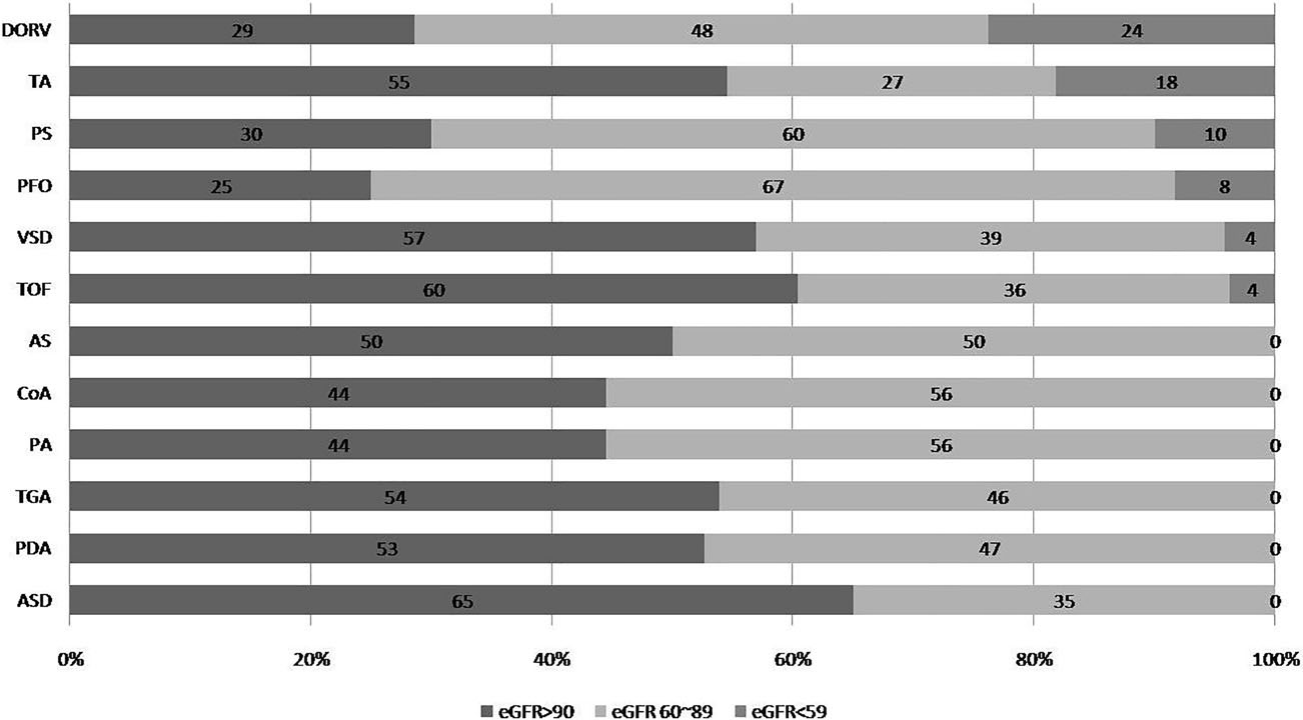
CKD incidence in CHD types. AS aortic stenosis, ASD atrial septal defect, CHD congenital heart disease, CKD chronic kidney disease, CoA coarctation of aorta, DORV double outlet right ventricle, PA pulmonary atresia, PDA patent ductus arteriosus, PFO patent foramen ovale, PS pulmonary stenosis, TA truncus arteriosus, TGA transposition of the great arteries, TOF tetralogy of Fallot, VSD ventricular septal defect

**Fig. 3:**
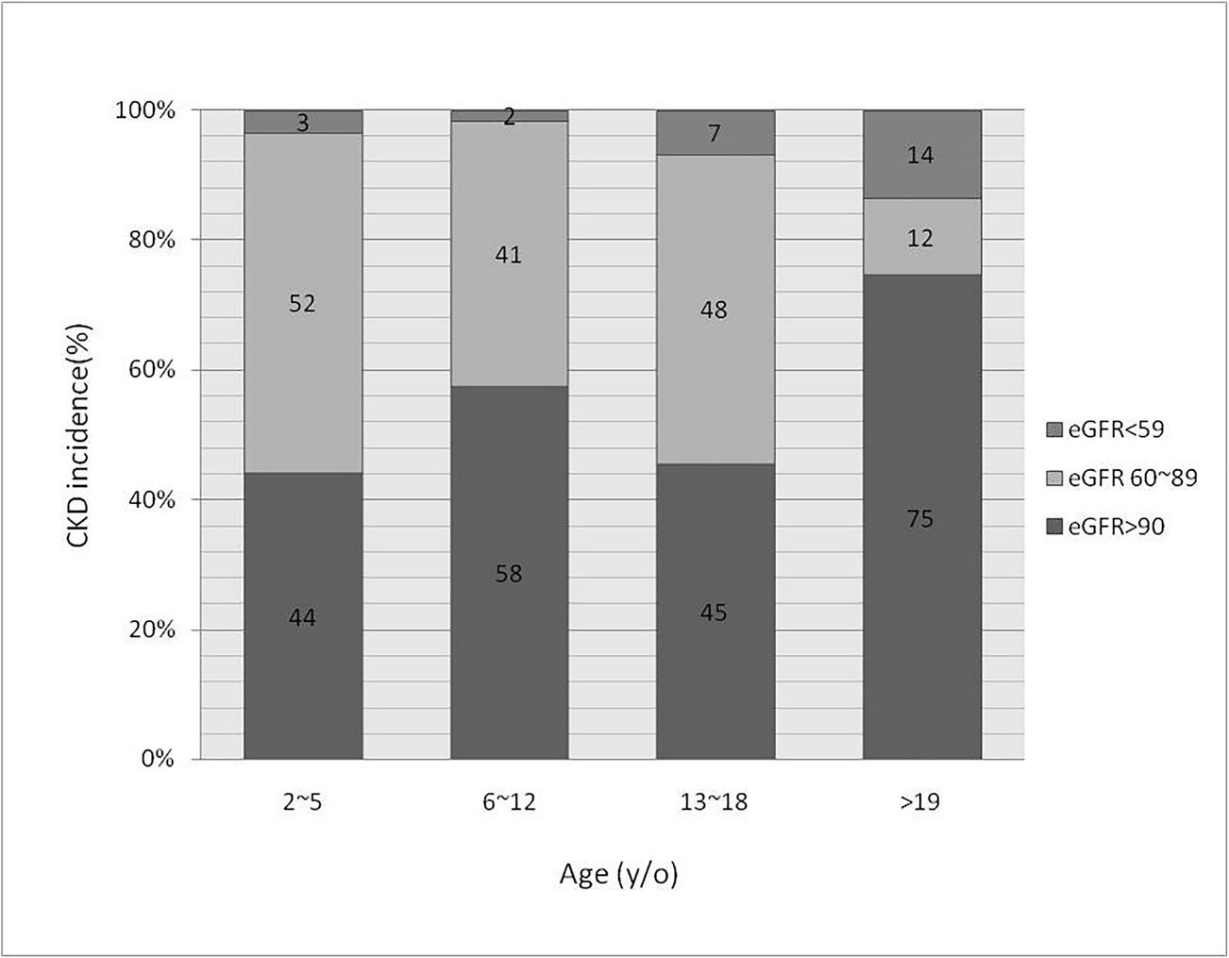
CKD incidence in each CHD age range. CHD congenital heart disease, CKD chronic kidney disease, eGFR estimated glomerular filtration rate

Overall, 167 (46.5%) patients with CHD developed CKD during follow-up; 18 (5%) met the criteria for CR-CKD (7 fulfilled the eGFR criterion, 13 fulfilled UPCR or UACR criteria, and 2 met both criteria). CR-CKD was most prevalent in the double outlet right ventricle (24%), followed by tricuspid atresia (18%), and PS (10%) groups (Fig. 2). On the other hand, NCR-CKD was most prevalent in VSD (21.4%), TOF (15.8%), ASD (12%), TGA (8.2%), and PS (5.6%). The mean CKD onset age was 14.0 ± 8.4 years. When we divided patients with CHD by age, CR-CKD was present in 3% of the preschool children (2–5 years old) and 2% in the school-age children (6–12 years old); this incidence increased to 7% in the adolescents (6–12 years old) and became most prominent (14%) in the young adults (older than 19 years) (Fig. 3).

### Risk factors for CKD in patients with CHD

The mean age of enrollment was higher in CR-CKD patients than in NCR-CKD patients (11.0 ± 7.7 and 6.4 ± 6.7, respectively). The mean follow-up duration for patients with CR-CKD and NCR-CKD were 5.8 ± 3.3 and 4.4 ± 3.5 years, respectively. In the CHD group, patients with cyanotic heart disease had a significantly higher CR-CKD incidence compared with those with acyanotic heart disease (61% and 37%, respectively, *P* = 0.042). We found no difference in the CR-CKD incidence in terms of PDA dependency, CHD severity, or type of heart failure. Regarding heart surgery, we found a significantly higher incidence of CR-CKD in those who received palliative heart surgery and a lower ratio of CR-CKD in those who received corrective heart surgery (*P* = 0.001; Table 1).

**Table 1:** Baseline characteristics of patients with CHD, *n* = 359

	Total	CR-CKD	NCR-CKD	
Variables	*n* = 359 (%)	*n* = 18 (%)	*n* = 341 (%)	*p* value
Age, years (mean ± SD)	6.6 ± 6.8	11.0 ± 7.7	6.4 ± 6.7	0.005
Male	195 (54%)	12 (67%)	183 (54%)	0.281
CCIS				0.203
0	314 (87%)	14 (78%)	300 (88%)	
≥ 1	45 (13%)	4 (22%)	41 (12%)	
CHD category				
Cyanotic CHD	138 (38%)	11 (61%)	127 (37%)	0.042
PDA-dependent	133 (37%)	4 (22%)	129 (38%)	0.181
Severe CHD	154 (43%)	11 (61%)	143 (42%)	0.109
Left-sided heart failure	150 (42%)	6 (33%)	144 (42%)	0.456
Operation				0.001
No need for operation	149 (42%)	7 (39%)	142 (42%)	
Corrective	156 (43%)	3 (17%)	153 (45%)	
Palliative	54 (15%)	8 (44%)	46 (13%)	
Image-related contrast exposure				0.008
0–1 (time)	242 (67%)	7 (39%)	235 (69%)	
≥ 2 (time)	117 (33%)	11 (61%)	106 (31%)	
Cardiac catheterization				0.738
0–1 (time)	242 (67%)	12 (67%)	214 (63%)	
≥ 2 (time)	133 (33%)	6 (33%)	127 (37%)	
Medications				
Anti-platelet	103 (29%)	5 (28%)	98 (29%)	0.930
ACEI and ARB	60 (17%)	2 (11%)	58 (17%)	0.513
Beta-blocker	62 (17%)	4 (22%)	58 (17%)	0.568
Loop diuretics	174 (49%)	4 (22%)	170 (50%)	0.022
Potassium-sparing diuretics	130 (36%)	3 (17%)	127 (37%)	0.077
Warfarin	27 (8%)	2 (21%)	25 (7%)	0.553
Digoxin	49 (14%)	1 (5%)	48 (14%)	0.305

*ACEI*, angiotensin-converting enzyme inhibitor; *ARB*, angiotensin receptor blocker; *CCIS*, Charlson comorbidity index score; *CHD,* congenital heart diseases; *CR-CKD*, clinically recognized chronic kidney disease; *NCR-CKD*, non-clinically recognized CKD; *PDA*, patent ductus arteriosus

During follow-up, 117 (33%) patients received two or more image-related contrast exposures (CT or MRI) of the brain, heart, chest, or abdomen. A significantly higher incidence of CR-CKD was noted in patients undergoing more contrast-enhanced imaging studies (*P* = 0.008), but a similar trend was not observed in patients receiving cardiac catheterizations.

Diuretics were the most commonly prescribed medications, with 174 patients (49%) receiving loop diuretics. Significantly fewer patients with CR-CKD than patients with NCR-CKD were prescribed diuretics (22% and 50%, respectively, *P* = 0.022). A similar trend was noted for potassium-sparing diuretics, although it did not reach significance. Of the 60 patients (17%) using ACEIs or ARBs, the difference between the two groups was nonsignificant (*P* = 0.513). No significant difference was observed in the use of anticoagulants or other medications between the two groups.

### Major determinants of CKD in patients with CHD

In univariate logistic regression, age > 18 years is an independent risk factor for CR-CKD (odds ratio (OR), 4.89 (1.86–12.82); *P* = 0.001; Table 2) and remained a major determinant after stepwise logistic regression analysis (adjusted OR, 5.59 (1.97–15.92); *P* = 0.001; Table 3). Palliative heart surgery was also a risk factor for CR-CKD (OR, 3.53 (1.21–10.26); *P* = 0.021) in univariate logistic regression. Corrective heart surgery was a protective factor for CR-CKD in stepwise logistic regression analysis (adjusted OR, 0.14 (0.03–0.59); *P* = 0.007). Cyanotic CHD was related to CR-CKD in univariate logistic regression (OR, 2.65 (1.01–7.00); *P* = 0.05), although it became insignificant in stepwise logistic regression analysis, there was a trend of high relationship in cyanotic CHD and CR-CKD (adjusted OR, 5.12 (0.82–32.57); *P* = 0.08). Loop diuretic usage and two or more image-related contrast exposures were minor factors relating to CR-CKD (OR, 0.29 (0.09–0.89) and 3.45 (1.31–9.24); *P* = 0.031 and 0.012, respectively.) Nonetheless, these factors became nonsignificant in stepwise logistic regression analysis.

**Table 2:** Univariate logistic regression analysis of CR-CKD in patients with CHD; *n* = 359

Variables	OR (95% CI)	*p* value
Age older than 18-years-old	4.89 (1.86–12.82)	0.001
Male	1.73 (0.63–4.71)	0.286
CCIS index >0	2.09 (0.66–6.66)	0.212
CHD category		
Cyanotic CHD	2.65 (1.01–7.00)	0.050
PDA-dependent	0.47 (0.15–1.46)	0.191
Severe CHD	2.18 (0.82–5.75)	0.117
Left-sided heart failure	0.68 (0.25–1.87)	0.458
Operation		
No need for operation	1	
Corrective	0.40 (0.10–1.57)	0.117
Palliative	3.53 (1.21–10.26)	0.021
≥ 2 times image-related contrast exposure	3.48 (1.31–9.24)	0.012
≥ 2 times cardiac catheterization	0.84 (0.31–2.30)	0.738
Medications		
Anti-platelet	0.95 (0.33–2.75)	0.930
ACEI and ARB	0.61 (0.14–2.73)	0.517
Beta-blocker	1.39 (0.44–4.39)	0.570
Loop diuretics	0.29 (0.09–0.89)	0.031
Potassium-sparing diuretics	0.34 (0.10–1.19)	0.090
Warfarin	1.58 (0.34–7.26)	0.557
Digoxin	0.36 (0.05–2.76)	0.325

*ACEI*, angiotensin-converting enzyme inhibitor; *ARB*, angiotensin receptor blocker; *CCIS*, Charlson comorbidity index score; *CHD*, congenital heart disease; *CI*, confidence interval; *CR-CKD*, clinically recognized chronic kidney disease; *OR*, odds ratio; *PDA*, patent ductus arteriosus

**Table 3:** Stepwise logistic regression analysis of CR-CKD in patients with CHD; *n* = 359

Variables	aOR (95% CI)	*p* value
Age older than 18 years	5.59 (1.97–15.92)	0.001
Cyanotic CHD	5.12 (0.82–32.57)	0.080
Corrective operation	0.14 (0.03–0.59)	0.007
Palliative operation	1.61 (0.23–11.31)	0.632

*aOR*, adjusted odds ratio; *CI*, confidence interval; *CHD,* congenital heart disease; *CR-CKD*, clinically recognized chronic kidney disease

## Discussion

Our study revealed a high CKD incidence in patients with CHD compared to the general population. The onset of CR-CKD occurred mostly during adolescence and young adulthood, but it may occur as early as childhood. Early corrective surgery for CHD may be a protective factor in the development of CR-CKD.

CKD is defined by Kidney Disease: Improving Global Outcomes (KDIGO) practice guidelines as persistent kidney damage or decreased kidney function for 3 months or more. Kidney damage indicators include appearance on medical imaging, pathological anomalies from a kidney biopsy, urinary examination anomalies, and decreased kidney function (defined as decreased eGFR calculated by population-appropriate formulas) [8]. Pediatric CKD is underdiagnosed and heterogenous among studies because of the insidious nature of early-stage CKD and the different definitions of pediatric CKD across studies. Currently, the most thorough study on the incidence of pediatric CKD stages 2–5 identified a range of 7.7 to 12.1 per million age-related population [9], compared to the 5% CR-CKD incidence in our study. The incidence of CKD in patients with CHD may be 413-fold to 650-fold higher than that of the general pediatric population.

Our study demonstrated that age is an independent risk factor for CKD in patients with CHD. CR-CKD was noted in 2–3% of patients with CHD during childhood, and the incidence increased to 7–14% during adolescence and young adulthood. As children age, the incidence and prevalence of CKD increases. Similar findings have been noted over time in adults with CHD. A study including 1102 adult patients with CHD (mean age 36.0 ± 14.2 years, 9% had eGFR <60 mL/min/1.73 m^2^) yielded an 8.3 mL/min/1.73 m^2^ (7.3–9.4 mL/min/1.73 m^2^) decrease in eGFR per 10-year increase in age [2]. Kidney dysfunction in adults with CHD has drawn the attention of physicians because it is associated with increased long-term mortality [2, 3, 10]. However, in our study, CR-CKD was detected in 2-year-old patients with CHD. Another study of 94 children with cyanotic heart disease identified an instance of a 1-month-old infant developing microalbuminuria [11]. Thus, patients with CHD should be considered at risk for CKD. Physicians should carefully monitor kidney function in children with CHD because rapid intervention and treatment for CKD may slow the deterioration of kidney function and improve long-term survival.

Several mechanisms of CKD pathogenesis in CHD have been proposed. Patients with cyanotic heart disease have chronic hypoxia, which increases blood viscosity, subsequently increasing efferent arteriole tone and the filtration fraction [12] and resulting in glomerular hyperfiltration and sclerosis. Another mechanism of kidney dysfunction in patients with CHD is low cardiac output resulting in kidney hypoperfusion. This leads to the activation of the renin–angiotensin–aldosterone system and a subsequent increase in sodium and water retention, with the system inducing left ventricular remodeling and a further reduction of cardiac output [13]. Other important factors were recurrent episodes of ischemia and acute kidney injury (AKI) during and after cardiac surgery, with cardiopulmonary bypass in patients with CHD undergoing open-heart surgery. Open-heart surgery and AKI coincide in 30–50% of cases, and the 5-year cumulative CKD rate in a previous study was four times higher in patients with AKI during open-heart surgery than it was in those without kidney injury [14]. Nephrotoxic drugs such as nonsteroidal anti-inflammatory drugs and diuretics often prescribed for CHD are other potential factors. We found that cyanotic CHD was a risk factor for CKD. However, corrective heart surgery for CHD prevented CKD; this benefit may be as large as a 7-mL/min/1.73 m^2^ increase in eGFR compared with CHD patients who underwent palliative heart surgery [2]. The correction of an abnormal cardiac structure may improve systemic cyanosis and cardiac output, reduce local kidney tissue hypoxia, and improve hypoperfusion. Therefore, early corrective heart surgery is crucial. Whether CHD is correctable depends not only on the surgeon’s technique or the willingness of the patient and their family but also on the condition of the heart disease. Therefore, CHD correctability is not always a modifiable risk factor for CKD in patients with CHD.

A potential modifiable risk factor in patients with CHD for the prevention of CKD development is the contrast exposures used during imaging examinations. Iodinated contrast media can induce AKI. In the general population without comorbidities, the risk of contrast-induced AKI may be less than 1% [15], but studies have shown that older adults and patients with congestive heart failure, diabetes mellitus, or volume depletion have an increased contrast-induced nephropathy risk of 25% [16]. Patients with CHD are often predisposed to contrast-induced AKI because of complications of congestive heart failure or because they receive fluid restriction therapy to control left-sided heart failure. Therefore, to prevent repetitive AKI and subsequent CKD in patients with CHD, a discreet decision should be made before contrast-related imaging studies.

Diuretic usage is associated with a decreased eGFR and increased incidence of albuminuria in patients with CHD [2, 3, 17]. Although it is used for controlling congestive heart failure in patients with CHD, loop diuretic usage in patients with non-CR-CKD was higher than in those with CR-CKD in the present study. This may be related to a higher rate of left-sided heart failure in patients with non-CR-CKD, but no differences were observed regarding other medications (e.g., ACEIs, ARBs, beta-blockers, or digoxin) commonly prescribed for congestive heart failure. We were unable to explain the contradictory result in the present study from our current analysis; therefore, a further study with a larger sample size may clarify this result.

Our study had some limitations. First, this is a retrospective study, therefore, 1352 of the 2172 patients with CHD in our database were excluded because of the lack of relevant serum creatinine or UPCR / UACR readings between 2010 and 2019. A further cohort study with regular follow-up intervals may comprehensively describe the CKD incidence in patients with CHD. Second, repetitive AKI was a potential factor for the development of CKD; however, we were unable to identify AKI episodes in our cohort from the current database. Third, patients younger than 2 years old with CHD were excluded from our study because their eGFRs were usually lower than 90 mL/min/1.73 m^2^ and, even with correction for body surface area, they were unsuitable for KDIGO CKD staging. Because CKD can still occur in this age group, a further classification system for this particular age range may help in the diagnosis of CKD in patients less than 2 years old.

## Conclusion

CHD patients have a high incidence of CKD, and the development of CKD may be as early as childhood. The early detection of CKD and prompt corrective heart surgery for CHD, and decreased contrast exposure during follow-up may be beneficial for kidney function.

## Data Availability

The datasets generated during and/or analyzed during the current study are available from the corresponding author on reasonable request.
